# Entrectinib-Induced Heart Failure in a Patient With Metastatic Lung Adenocarcinoma: A Case Report

**DOI:** 10.7759/cureus.32174

**Published:** 2022-12-04

**Authors:** Yukiko Otsu, Yuki Kata, Hirokazu Takayasu, Satoshi Inoue, Takeshi Kaneko

**Affiliations:** 1 Department of Respiratory Medicine, Yamato Municipal Hospital, Yamato, JPN; 2 Department of Pulmonology, Yokohama City University Graduate School of Medicine, Yokohama, JPN

**Keywords:** non small cell lung cancer, ros-1 rearrangement, entrectinib, heart failure, chemotherapy associated cardiotoxicity

## Abstract

Entrectinib is a recently approved multikinase inhibitor to treat advanced c-ros oncogene1 (ROS1) positive non-small cell lung cancer (NSCLC). Although molecular targeted therapy is generally well tolerated, cardiovascular adverse events have been described in recent years. We report a case of NSCLC with ROS1 rearrangement where the patient developed drug-induced heart failure after receiving entrectinib.

A 74-year-old non-smoker female patient was diagnosed with stage IVB lung adenocarcinoma with ROS-1 positive and right breast cancer stage I. We started on entrectinib as the first-line therapy for lung cancer. Five days after, she developed oral dysesthesia and blood creatinine increased. These findings gradually worsened, so we temporarily discontinued entrectinib. After withholding the drug for 14 days, these findings improved, and we resumed entrectinib at a reduced dose. On day 19 of the reduced entrectinib dose, she presented to the outpatient with shortness of breath and bilateral lower extremity edema, accompanied by respiratory failure. Laboratory evaluation revealed elevated N-terminal pro-brain natriuretic peptide (NT-pro BNP), troponin I, creatine kinase (CK), and C reactive protein (CRP), and transthoracic echocardiogram showed congestive heart failure (CHF) with a preserved ejection fraction (HFpEF). She did not complain of chest pain and fever, so we did not consider ischemic heart disease and viral myocarditis in the initial evaluation. There was no other causative cause of CHF. Therefore, we suspected entrectinib-related heart failure. Her symptoms improved and she recovered her cardiac function to baseline within a week of discontinuation of entrectinib and standard heart failure treatment. She developed heart failure after a one-step dose reduction and was prone to cardiotoxicity due to entrectinib. Considering that she could be treated with crizotinib, we decided discontinuation of entrectinib permanently. This case report highlights the potential cardiotoxicity of entrectinib and suggests the need for close monitoring of the cardiac functions of patients receiving entrectinib.

## Introduction

Advances in molecular profiling have identified multiple targetable mutations or gene rearrangements in lung adenocarcinoma. Molecularly targeted therapies have advanced the treatment of advanced non-small cell lung cancer (NSCLC) [1]. The c-ros oncogene1 (ROS1) fusion genes are identified in approximately 1%-2% of NSCLC patients [2]. Entrectinib, a relatively new tyrosine kinase inhibitor (TKI), has been reported to be effective as primary therapy for ROS1 fusion-positive NSCLC [3]. Although molecular targeted therapy is generally well tolerated, cardiovascular adverse events such as arrhythmia, heart failure, myocardial infarction, QTc prolongation, myocarditis, and pericarditis have been described in recent years [4]. Here, we report a patient with ROS1 fusion gene-positive lung cancer who developed drug-induced heart failure after receiving entrectinib.

## Case presentation

**Figure 1:**
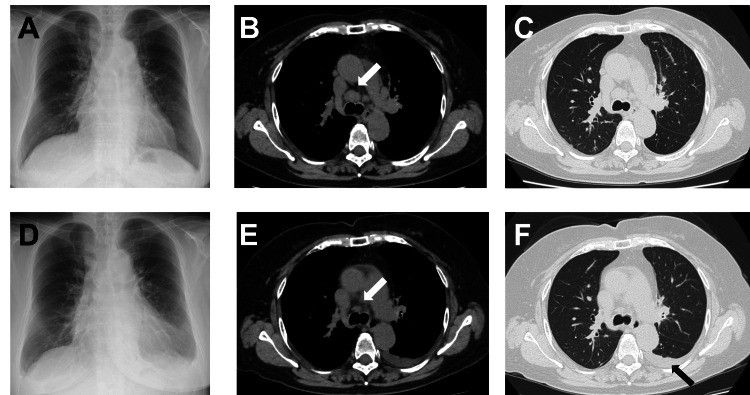
Chest X-ray and computed tomography (CT) findings before entrectinib therapy (A, B, and C) and after entrectinib therapy (D, E, and F) A: Chest X-ray showed an enlarged mediastinal shadow; B and C: Chest CT revealed multiple enlarged mediastinal lymph nodes; D: Chest X-ray showed a left pleural effusion after entrectinib treatment; E and F: Chest CT showed the reduction of multiple enlarged mediastinal lymph nodes and a new onset of minimal left-sided pleural effusion that was not present in the previous CT (arrows)

A 74-year-old female was referred to our hospital in March 2022 after a chest computed tomography (CT) for a thorough examination of a dry cough which revealed multiple enlarged mediastinal lymph nodes (Figure 1). She was a non-smoker and had a medical history of dyslipidemia, bronchial asthma, and sigmoid colon cancer. 18F-fluorodeoxyglucose positron emission tomography/computed tomography (18F-FDG PET/CT) showed a mass in the left upper lobe of the lung (considered the primary lesion), multiple enlarged mediastinal and hilar lymph nodes, enlarged right breast, and multiple bone metastases. A surgical biopsy was performed for mediastinal lymphadenopathy, and histopathological examination revealed mediastinal lymph nodes metastasis of lung adenocarcinoma. She was diagnosed with ROS1 rearrangement by next-generation sequencing - the Oncomine Dx Target Test Multi-CDx system (Thermo Fisher Scientific Waltham, MA). We also performed a right breast mass needle biopsy and diagnosed invasive ductal carcinoma. We staged the breast tumor as right breast cancer stage I (T1N0M0) based on the absence of axillary lymph node metastasis and the low probability of distant metastasis. On the other hand, we finally diagnosed her lung tumor as lung adenocarcinoma and staged stage IVB (T1bN3M1c) due to multiple bone metastases. 

We considered that the prognosis depended on lung cancer rather than breast cancer and started treatment with entrectinib 600 mg orally once daily as the first-line treatment in May 2022. On day five after entrectinib administration, she developed oral dysesthesia (grade 1 according to Common Terminology Criteria for Adverse Events [CTCAE] version 5.0), and her blood creatinine increased (grade 2). These findings gradually worsened, so we temporarily discontinued entrectinib. On withholding the drug for 14 days, these improved, and treatment with entrectinib was resumed at a reduced dose of 400 mg orally once daily.

On day 19 of the reduced entrectinib dose, she presented to the outpatient unit with shortness of breath and bilateral lower extremity edema, accompanied by respiratory failure (heart rate 67 bpm, blood pressure 125/78 mmHg, SpO2 87% in ambient air) and was admitted to our hospital. Laboratory evaluation revealed elevated N-terminal pro-brain natriuretic peptide (NT-pro BNP), troponin I, creatine kinase (CK), and C reactive protein (CRP) (Table 1). An electrocardiogram (ECG) showed normal sinus rhythm without ischemic changes or arrhythmia. Chest X-ray showed enlargement of the heart and a left-sided pleural effusion (Figure 1). Transthoracic echocardiogram (TTE) showed the left ventricular ejection fraction (LVEF) was mildly decreased to 61% compared to that before entrectinib treatment (13% points down). In addition, it showed that the average mitral E/e' ratio was 18, septal e' velocity was 4.1 cm/sec, and tricuspid regurgitant velocity (TRV) was 2.7 m/sec. The TTE findings and BNP level (614.50 pg/mL) met the diagnostic criteria of heart failure with a preserved ejection fraction (HFpEF) as per the American Society of Echocardiography and the European Association of Cardiovascular Imaging recommendations [5]. We did not consider ischemic heart disease and viral myocarditis in the initial evaluation because she did not complain of chest pain and fever. Therefore, we suspended coronary angiograms and the evaluation of viral titers. She had no valvular heart disease that could cause heart failure and was not receiving any new medications other than entrectinib. A chest CT revealed decreased left pulmonary upper lobe mass, mediastinal lymph nodes, and a new onset of pleural effusion on the left that was not present in the previous CT (Figure 1). The left pleural effusion was too small to aspirate, so we did not submit it for analysis. Her serum carcinoembryonic antigen (CEA) level decreased. These results suggested that entrectinib significantly reduced tumors but caused grade 3 heart failure.

**Table 1:** Clinical presentation and hematological parameters SpO2: oxygen saturation; AST: aspartate aminotransferase; ALT: alanine aminotransferase; LDH: lactate dehydrogenase; Na: sodium; K: potassium; Cre: creatinine; CK-MB: creatine kinase-MB; BUN: blood urea nitrogen; eGFR: estimated glomerular filtration rate; Hb: hemoglobin; WBC: white blood cells; Plt: platelet count; CRP: C-reactive protein; NT-proBNP: N-terminal pro-brain natriuretic peptide.

	Before entrectinib	On admission	At discharge
SpO_2_(%)(ambient air)	97	87	97
AST(U/L)	16	22	20
ALT(U/L)	11	14	12
LDH(U/L)	214	247	218
Na(mEq/L)	141	144	144
K(mEq/L)	4.1	4.7	4.8
Cre(mg/dL)	0.58	0.93	0.76
CK(U/L)	61	164	108
CK-MB(ng/mL)	NA	4.9	1.9
BUN(mg/dL)	16	19	16
eGFR	75.6	45.1	56.3
Hb(g/dL)	13.4	12.2	12.7
WBC(/µL)	7800	4600	4200
Plt(10^4^/µL)	34.7	43	41.6
CRP(mg/dL)	0.45	0.17	0.1
Troponin I(pg/mL)	NA	143	56
NT-proBNP(pg/mL)	55.6	614.5	178.1
NA indicates not assessed.			

**Figure 2:**
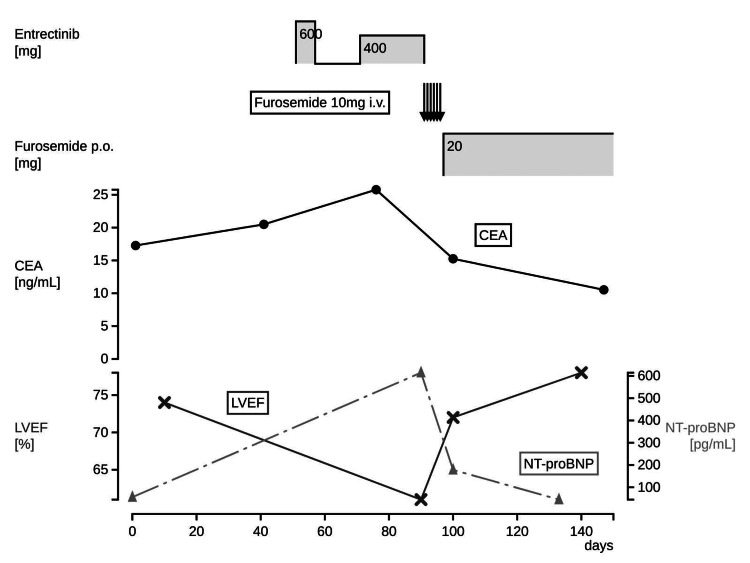
Clinical course CEA: carcinoembryonic antigen; NT-proBNP: N-terminal pro-brain natriuretic peptide; LVEF: left ventricular ejection fraction.

Entrectinib therapy was again interrupted, and intravenous furosemide 10 mg daily was administered. After six days of this treatment, her serum CK and troponin I level decreased (Table 1). In addition, TTE revealed restoration of LVEF up to 72%. Recovering from heart failure, she was discharged from our hospital with oral furosemide 20 mg daily. Her cardiac function improved to baseline in about one week after entrectinib suspension (Figure 2). Post-discharge imaging studies showed a decreased left-sided pleural effusion. We suspected that the pleural effusion was unilateral but due to cardiac failure since it improved with the discontinuation of entrectinib and administration of diuretics and did not relapse. After consulting with cardiologists, we decided not to initiate cardioprotective therapies: angiotensin-converting enzyme (ACE) inhibitors, angiotensin Ⅱ receptor blockers (ARBs), and beta-blockers because there was no evidence of their efficacy on HFpEF and she did not have hypertension [6]. She developed grade 3 heart failure after a one-step dose reduction, and we determined that she was prone to cardiotoxicity due to entrectinib. Considering that she could be treated with crizotinib; we decided to discontinue entrectinib treatment permanently.

## Discussion

Entrectinib is an oral multikinase inhibitor that potently and selectively inhibits ROS1 (encoded by the c-ros oncogene 1), the tropomyosin receptor kinase (TRK) family including TRKA, TRKB, and TRKC proteins (encoded by neurotrophic TRK (NTRK) 1, NTRK2, and NTRK3 genes), and anaplastic lymphoma kinases (ALK). It suppresses the growth of malignant tumors with NTRK1/2/3, ROS1, or ALK gene fusion mutations. It is proven to be effective for brain metastases because it can penetrate the blood-brain barrier and remain within the central nervous system [7]. The Food and Drug Administration (FDA) (United States), European Medicines Agency (EMA) (European Union), and Pharmaceuticals and Medical Devices Agency (PMDA) (Japan) approved it in 2019-2020 for the treatment of ROS1 fusion gene-positive metastatic NSCLC in adults who have not previously received a ROS1 inhibitor in 2019-2020 [8]. In an integrated analysis of three phase I/II trials (ALKA-372-001, STARTRK-1, and STARTRK-2), the overall response rate (ORR) was 68%, and median progression-free survival (PFS) was 15.7 months in ROS1-positive NSCLC. There were 224 patients in total included in the overall ROS1 fusion-positive NSCLC safety-assessable population. Treatment-related adverse events (TRAEs) were reported in 211 patients (94%), and approximately half of them were grade 1 or 2 (CTCAE). The common grade 1 to 2 TRAEs were dysgeusia, dizziness, and constipation; the most common grade 3 TRAE was increased weight. Cardiac disorders occurred in 11 patients (4.9%), eight of whom were grade 3 or 4. Twelve patients (5%) discontinued entrectinib because of TRAEs. The most common TRAEs causing discontinuation of entrectinib were cardiac disorders (n = 4; 2%) [3]. According to the FDA drug prescribing recommendations, among the three phase I/II trials, congestive heart failure (CHF) occurred in 3.4% of patients, including grade 3 (2.3%) and one patient (0.3%) who developed grade 4 myocarditis. Taking this into account, they warned that entrectinib may cause CHF or make the CHF that patients already have worse. They also mentioned that the median time of the first onset of CHF in clinical trials was two months (ranging from 11 days to 12 months), occurring relatively early in treatment [9]. To date, only one real-world case has been reported as cardiovascular adverse events of entrectinib; a 51-year-old female with ROS-1 positive NSCLC suffered from myocarditis [10].

Several targeted therapies used in NSCLC are known to cause cardiotoxicities, such as crizotinib and alectinib, ALK inhibitors, with conduction disease and osimertinib, epidermal growth factor receptor (EGFR) inhibitors, with QT prolongation and heart failure [4]. The European Society of Cardiology (ESC) published the recent international definitions of cancer therapy-related cardiovascular toxicities in August 2022. This guideline recommends that myocardial infarction, cardiomyopathy, and heart failure due to cancer treatment are described as cancer therapy-related cardiac dysfunction (CTRCD). CTRCD is classified into seven categories according to symptoms' presence or absence and severity. The appropriate time to consider cardiovascular toxicity prophylaxis in cancer patients is at the time of cancer diagnosis and before the initiation of cancer treatment. In the ESC guidelines, baseline cardiovascular risk assessments such as physical examination, blood pressure measurement, ECG, TTE, lipid profile, and HbA1c measurement, are recommended in patients before ALK inhibitors and EGFR inhibitors [11]. In our case, the patient had a medical history of dyslipidemia, but there were no abnormalities in the laboratory test and ECG before entrectinib initiation. The TTE findings before treatment showed that LVEF was preserved, but we did not scrutinize the presence of diastolic dysfunction. We supposed that her cardiotoxicity risk was low, but she developed CHF requiring hospitalization. On admission, troponin I was elevated without symptoms of chest pain and ischemic changes on ECG, so we did not suspect acute coronary syndromes proactively. We could not rule out the possibility that the patient had asymptomatic HFpEF before entrectinib treatment. However, even if she did not develop HFpEF anew, she at least exacerbated her cardiac function from baseline. Furthermore, she developed CHF after initiation of entrectinib, had no other cause for CHF, and then her cardiac function improved with entrectinib suspension. Consequently, we diagnosed her with entrectinib-related severe CTRCD.

There is no specific treatment for CTRCD. According to the ESC guidelines, treating acute and chronic heart failure following recent general heart failure guidelines is recommended [11]. Given the patient's hemodynamic stability at presentation, we decided to start intravenous loop diuretics. For oncological strategy about entrectinib, according to the prescribing information approved by FDA, when considering cardiovascular adverse events regardless of the severity, they firstly recommend that physicians temporarily withhold entrectinib until recovery to less than or equal to grade one. They recommend resuming treatment with a one-step dose reduction or permanent discontinuation [9]. We decided not to rechallenge entrectinib, given that our patient developed CTCRD after a one-step dose reduction and was prone to cardiotoxicity due to entrectinib.

## Conclusions

We presented a case of symptomatic CTRCD after entrectinib treatment in a patient with ROS1-rearranged metastatic lung adenocarcinoma. Physicians should monitor cardiac function as patients may develop heart failure, especially in the early phase of entrectinib initiation. The number of cases where entrectinib is prescribed is small, and the frequency of cardiotoxicity and its severity in the real world is still not well understood. Further research is needed to define the management of entrectinib-induced cardiotoxicity.
